# Advancing therapeutic target discovery in pulmonary diseases: evolution and application of space exploration technologies

**DOI:** 10.3389/fcell.2025.1657720

**Published:** 2025-09-18

**Authors:** Bo Yang, Quan Zheng, Guozheng Li, Bingnan Liao, Zhongxiao Lin, Hao He

**Affiliations:** ^1^ Guangzhou Municipal and Guangdong Provincial Key Laboratory of Molecular Target and Clinical Pharmacology, The NMPA and State Key Laboratory of Respiratory Disease, School of Pharmaceutical Sciences, Guangzhou Medical University, Guangzhou, China; ^2^ Zhengda Tianqing Pharmaceutical Group Co., LTD, Lianyungang, China; ^3^ School of Pharmacy, Macau University of Science and Technology, Macau, China; ^4^ Shenzhen Longgang Second People’s Hospital, Shenzhen, Guangdong, China

**Keywords:** epigenetics, pulmonary diseases, chromatin structure, Hi-C technology, therapeutic targets

## Abstract

Epigenetic alterations are associated with various pulmonary diseases. In recent years, the concept of epigenetic inheritance influenced by spatial variations has garnered increasing attention. Alterations in three-dimensional (3D) chromatin architecture have been demonstrated to play a crucial role in regulating gene expression and influencing the pathogenesis and progression of lung-related diseases. Techniques such as high-throughput chromosome conformation capture (Hi-C) have emerged as powerful tools for detecting spatial chromatin conformational changes. In this review, we summarize key targets identified through Hi-C and related methodologies in the context of pulmonary diseases and explore their potential implications for epigenetic therapies.

## 1 Introduction

Pulmonary disease, which encompass a variety of conditions such as chronic obstructive pulmonary disease (COPD), asthma, pulmonary fibrosis, and lung cancer, have become significant contributors to mortality and health burdens worldwide. The development of these diseases is influenced by a multitude of factors, including genetics, environmental exposures, lifestyle choices, and immune responses (Wang et al., 2025). Increasing research indicates that epigenetics plays a crucial role in the pathogenesis of pulmonary disease. Epigenetics refers to heritable changes in gene expression that do not involve alterations to the DNA sequence itself, but rather are mediated through mechanisms such as DNA methylation, histone modifications, and the action of non-coding RNAs (Casas-Recasens et al., 2024).

Environmental factors, including air pollution, tobacco smoke, and occupational exposures, can induce epigenetic changes that affect gene expression. For instance, certain pollutants can lead to inflammatory responses in the lungs and promote the development of chronic pulmonary disease by altering the methylation status of relevant genes (Jurkowska, 2024). Additionally, genetic factors may influence individual responses to environmental triggers through various epigenetic mechanisms, making certain populations more susceptible to pulmonary disease. The importance of epigenetic interventions in lung disease research is becoming increasingly evident (Ritzmann et al., 2025). By modulating the epigenetic states of specific genes, novel therapeutic strategies can be developed. For example, inhibitors targeting specific histone deacetylases (HDACs) or DNA methyltransferases may play a significant role in controlling excessive inflammatory responses or the progression of fibrosis in the lungs. These interventions could not only improve the effectiveness of existing treatments but also provide preventive opportunities for individuals at high risk who have not yet developed disease (Klibaner-Schiff et al., 2024).

With advancements in technology, research tools in epigenetics, such as chromatin immunoprecipitation sequencing (ChIP-seq), assay for transposase-accessible chromatin using sequencing (ATAC-seq), DNA methylation sequencing (methyl-seq), and transcriptome sequencing (RNA-seq) (Goodwin et al., 2016), allow for a deeper understanding of the epigenetic mechanisms underlying pulmonary disease. These technologies pave the way for the identification of new biomarkers and therapeutic targets, promoting the advancement of personalized medicine. For example, analyzing the epigenetic landscape of patients with pulmonary disease enables researchers to identify specific epigenetic alterations and design targeted interventions accordingly. The advent of 3D structural detection technologies, such as Hi-C, has provided researchers with novel perspectives, thereby enabling the investigation of the complexities of gene expression regulation from the standpoint of 3D genomic architecture (van Berkum et al., 2010). The importance and significance of Hi-C technology in lung disease research are reflected in several aspects.

First, Hi-C and relative technologies capture the 3D structure of chromatin, revealing the interactions between long-range regulatory elements and their target genes within the genome. This spatial relationship is often difficult to detect through traditional sequence analysis; however, in the context of pulmonary disease, the spatial relationships among different genes and regulatory elements often directly influence the onset and progression of these conditions. For example, in cases of COPD or lung cancer, specific enhancers may promote the expression of precursor genes through 3D interactions, and 3D-caputre technology help researchers identify these critical regulatory elements, thereby providing new strategies for targeted therapies (Yoon et al., 2024; Guo et al., 2024). Besides, the 3D-caputre technology also plays a vital role in the discovery of biomarkers. By mapping the epigenetic landscapes of patients with pulmonary disease, researchers can uncover specific changes in 3D genomic structures associated with these diseases. These alterations can serve not only as biomarkers for early diagnosis but also hold potential as key indicators for disease prediction and prevention in personalized medicine (Ritzmann et al., 2025; Martino et al., 2023). For instance, if a particular genomic structural change is prevalent in the majority of lung cancer patients, it may become an effective target for precise therapeutic interventions. Additionally, the complex mechanisms of pulmonary disease require multiple research perspectives, and epigenetic interventions offer new hope for the development of novel therapies, the timeliness and high-throughput nature of 3D-caputre technology enhance its feasibility for target discovery applications (Zhang et al., 2024).

**FIGURE 1:**
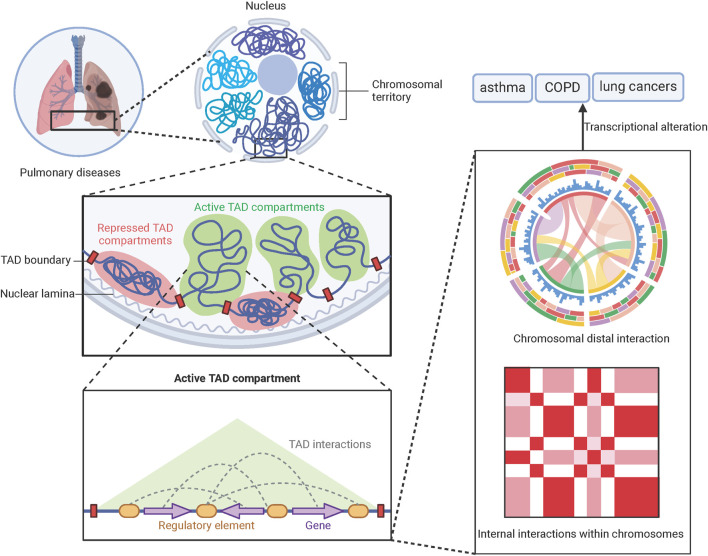
Hi-C-based therapeutic targets discovery in pulmonary disease. At the nuclear level in pulmonary diseases, chromatin is organized into spatially segregated compressed and transcriptionally active compartments. Within these active compartments, numerous regulatory elements orchestrate gene expression programs that drive disease phenotypes. Through three-dimensional genomic profiling, we can systematically map long-range chromatin interactions at nucleotide resolution. This approach enables mechanistic dissection of pathogenic processes in asthma, COPD, and lung cancer, while simultaneously facilitating the identification of novel therapeutic targets.

By better understanding and modulating the epigenetic changes associated with pulmonary diseases, this review will summarize the integration of Hi-C and related data in pulmonary diseases (COPD, asthma, pulmonary fibrosis, and lung cancer) (Figure 1), and discuss whether the deeper applications of Hi-C in exploring the pathogenesis of pulmonary diseases.

## 2 The history and the usage of Hi-C-based technology

In the 1980s and 1990s, scientists achieved a comprehensive understanding of the linear sequences of genomes; however, research on the spatial organization of chromosomes within the cell nucleus and their interactions was comparatively limited (Shendure et al., 2017). To explore these relationships, researchers began to develop novel experimental techniques. A pivotal study in 2002 introduced the 3C (Chromosome Conformation Capture) technology, pioneered by Job Dekker and others, aimed at investigating the 3D conformation of genomes by analyzing physical contact points between chromosomes (Dekker et al., 2002). This approach involved using crosslinkers, such as formaldehyde, to link interacting DNA fragments, followed by digestion and PCR amplification, which facilitated the capture of specific chromosomal interactions. Nonetheless, the 3C method was constrained by the size of the interacting fragments and could not be directly employed to survey fragments across the entire genome.

Subsequent advancements led to the development of several derivative techniques, including 4C (Chromosome Conformation Capture-on-Chip), which examines neighboring genomic regions of particular genes, and 5C (Chromosome Conformation Capture Carbon Copy), which is appropriate for large-scale analysis of interactions among multiple DNA fragments. Hi-C, an extension of 3C technology, merges chromosome conformation capture with high-throughput sequencing. In this technique, DNA is first treated with formaldehyde to crosslink it with proteins and stabilize its conformation, followed by digestion, biotin incorporation, ligation, and extraction. The processed nucleic acids undergo library construction and high-throughput sequencing, ultimately providing interaction information between chromosomal fragments through the analysis of sequencing data. This method assesses relationships in spatial positioning throughout the entire chromatin within the genome (Jiang et al., 2024).

Hi-C technology not only facilitates the study of interactions between chromosomal segments and the modeling of genomic folding, but it also has applications in genome assembly, haplotype mapping, and aiding in metagenome assembly (Jiang et al., 2024). Furthermore, Hi-C can be integrated with data from RNA-Seq and ChIP-Seq to elucidate the mechanisms underlying the formation of organismal traits, informed by gene regulatory and epigenetic networks (Lan et al., 2012). Consequently, detection technologies like Hi-C have opened new avenues for exploring the 3D architecture of chromatin, leading to investigations of 3D chromatin topologies in humans and mitochondria, as well as their implications for mitochondrial diseases (Feng et al., 2021; Kukat et al., 2015).

With the advancement of Hi-C technology and its derivatives, researchers are uncovering how genomic spatial organization influences gene expression regulation, chromosomal structure alterations, and their roles in diseases such as cancer and genetic disorders. These investigations have significantly enhanced our understanding of genomic biology and genetic regulatory mechanisms, proving valuable for elucidating disease pathogenesis and predicting disease occurrence. In addition, various related techniques have emerged based on Hi-C.

1. Capture Hi-C: Capture Hi-C technology enhances the conventional Hi-C library by introducing a specific probe-capture process (Martin et al., 2015). The fundamental principle involves designing probes targeted at specific regions, which are then used to isolate particular segments from conventional Hi-C libraries for sequencing. This technique is particularly advantageous for elucidating remote interactions in specified areas. The construction of the Capture Hi-C library is based on the conventional Hi-C method, with the addition of a step dedicated to capturing target segments from the obtained Hi-C library. The brief experimental workflow includes designing capture probes aimed at the regions of interest, which may be a specific area (e.g., a gene region of interest) or multiple regions (e.g., promoter regions). Following probe design, liquid-phase capture can be performed, ensuring that the adapter sequences at both ends of the Hi-C library are sealed to prevent erroneous captures associated with the adapter sequences. The capture probes are then utilized alongside the sealed Hi-C library, employing streptavidin magnetic beads to capture the Hi-C library in combination with the capture probes. Unbound Hi-C library fragments are subsequently washed away, and final PCR amplification produces the Capture Hi-C library suitable for sequencing.
2. Promoter Capture Hi-C: Promoter Capture Hi-C builds upon the conventional Hi-C library by adding a step specifically aimed at capturing promoter regions (Orlando et al., 2018). This technique focuses on interactions related exclusively to promoter regions, and as sequencing depth increases, the data derived from this region can significantly enhance, thereby improving the originally weak interaction signals for a more comprehensive analysis of promoter region interactions.
3. Diploid Chromatin Conformation Capture (Dip-C): Dip-C is a sequencing technique designed to study the 3D genome structure of single cells (Tan et al., 2018). Developed by Dr. Longzhi Tan and colleagues in 2018, this technique can produce high-resolution whole-genome 3D structures of individual human cells. Dip-C is currently the only method capable of measuring the 3D whole-genome structure of single cells at high resolution, achieving a resolution two orders of magnitude greater than traditional microscopic imaging methods, while also being user-friendly and cost-effective. The Harvard research team successfully utilized the Dip-C method to reconstruct the 3D structure of the human diploid single-cell genome from lymphoblastic cell lines and primary blood cells, revealing systematic differences between various cell types.
4. Significant progress has been made in single-cell Hi-C protocols. Gridina M *et al.* (2022) developed an ultra-high-resolution single-cell Hi-C method (Gridina et al., 2022). By improving cross - linking efficiency, more accurate capture of chromatin interactions at the single-cell level was achieved. In the amplification process, they minimized biases, which greatly enhanced the detection precision of chromatin interactions in individual cells. Li W *et al.* (2023) introduced a single-cell multi-omics integration technique, combining single-cell Hi-C with transcriptome analysis to reveal the association between 3D chromatin structures and gene expression at the single-cell level (Li et al., 2023). This helps understand how the spatial organization of chromatin influences gene transcriptional activity in individual cells, providing new avenues for exploring the complex regulatory mechanisms of cell-specific functions in pulmonary diseases.

Moreover, ChIA-Drop (Chromatin Interaction Analysis by Droplet) was developed as a polychromatin interaction analysis strategy (Kempfer and Pombo, 2020). This technique employs a 10X Genomics microfluidic system to isolate cross-linked and fragmented chromatin into microreactive droplets, each containing a unique barcode for detecting the DNA sequence of a single chromatin complex. Through this method, information regarding chromatin interactions can be obtained, addressing the limitations associated with Hi-C techniques in cell library construction. GAM infers chromatin interactions by capturing the co-segregation of genomic regions in nuclear sections through cryosectioning and laser microdissection (Beagrie et al., 2017). It does not require a ligation step and can detect complex chromatin contacts including multi-way interactions. This forms a complement to Hi-C, which relies on ligation, enriching our understanding of 3D genome structure. Similarly, SPRITE employs a split-pool barcoding strategy, performing multiple rounds of labeling on crosslinked chromatin complexes to identify higher-order chromatin interactions and associations with nuclear bodies (Quinodoz et al., 2018), and its ligation-independent nature enables it to capture long-range interactions that Hi-C might miss, further improve the Hi-C-based the 3D genom genome research system.

Currently, additional technologies derived from Hi-C include *in situ* Hi-C, DLO Hi-C, and HiChIP. (Mumbach et al., 2016; Lin et al., 2018; Rao et al., 2014). Collectively, these approaches provide profound insights into chromatin spatial organization and gene regulatory networks, while their convergence with experimental validation reveals mechanistic foundations of disease etiology.

## 3 The target discovery ability of Hi-C technology in pulmonary diseases

### 3.1 COPD and asthma

COPD is one of the leading causes of morbidity and mortality worldwide, imposing a substantial burden on the utilization of healthcare resources (Christenson et al., 2022). COPD is typically caused by long-term exposure to harmful particles or gases, with smoking being the primary risk factor. However, non-smoking-related factors such as air pollution, occupational exposure, and impaired lung development in early life also account for approximately half of the cases (Yang et al., 2022). The pathogenesis of COPD is complex, involving processes such as inflammation, oxidative stress, and airway remodeling. Although some progress has been made in understanding the disease mechanisms and identifying therapeutic targets in recent years, there is still a lack of breakthrough therapies that can significantly improve disease progression or reduce mortality (Rabe and Watz, 2017).

Asthma is a common chronic non-communicable disease characterized by variable respiratory symptoms and airflow limitation. Its pathogenesis involves complex interactions between genetic and environmental factors, leading to heterogeneous manifestations of airway inflammation and remodeling (Papi et al., 2018). However, traditional symptom-based management approaches are limited because there is no direct correlation between symptoms and airway inflammation or airflow limitation, resulting in suboptimal treatment outcomes (Shaw et al., 2021). In recent years, the development of biomarkers and biologics has provided new opportunities for phenotype-specific interventions in severe asthma, paving the way for personalized treatment. Moreover, acute asthma exacerbations represent a significant challenge in asthma management, with their pathogenesis closely related to type 2 airway inflammation. Novel targeted therapies have shown promising effects in this regard (Ramsahai et al., 2019). Guo et al. used Hi-C technology to analyze the chromosomal conformation of the 4q31 region associated with COPD susceptibility (Guo et al., 2024). They identified a distal enhancer that regulates the transcription of the Hedgehog-interacting protein (*HHIP*) gene in a SMAD3-dependent manner in human bronchial epithelial cells. The study also demonstrated that reduced expression of the *HHIP* gene leads to increased TGF β-induced epithelial-mesenchymal transition (*EMT*), which may be an important link in the pathogenesis of COPD.

Hi-C technology, by capturing spatial interactions of chromatin, has revealed the relationship between the 3D structure of the genome and gene expression regulation, providing a new perspective for elucidating the epigenetic mechanisms underlying pulmonary diseases. The pathogenesis of COPD and asthma is complex, involving interactions between multiple genetic and environmental factors. GWAS have identified several genetic loci associated with these two diseases, but the functions of these loci and their specific roles in disease are not yet fully understood. For example, a genetic locus on 17q12-q21 is significantly associated with childhood-onset asthma, while a region on 4q31 is closely linked to the susceptibility of COPD (Washington et al., 2022). However, most of these genetic loci are located in non-coding regions, and their functional studies require advanced genomic technologies. Mak et al., through whole-genome sequencing data combined with Hi-C analysis, identified a novel genetic association locus on chromosome 12 related to lung function (Mak et al., 2020). This locus is closely associated with the expression of the KIT ligand (*KITLG*, also known as *SCF*) gene, with the minor allele linked to increased expression of the *KITLG* gene in nasal epithelial cells. Moreover, the study found an interaction between the *KITLG* gene and exposure to air pollution, suggesting that *KITLG* may play a role in asthma pathogenesis by modulating allergic inflammation.

Stuart et al. utilized Hi-C technology combined with CRISPRi (CRISPR interference) to study the functions of non-coding regions associated with asthma, cystic fibrosis (CF), COPD, and idiopathic pulmonary fibrosis (IPF) (Stuart et al., 2020). They found that non-coding regions on 19q13, 17q21, and 11p15 interact with neighboring genes and may function as enhancers. Using CRISPRi, the researchers further validated the regulatory effects of these regions on gene expression, demonstrating the effectiveness of Hi-C technology in identifying potential gene regulatory elements. Additionally, Granell et al. used Hi-C technology to analyze genetic loci associated with persistent wheezing in childhood and identified a susceptibility locus near the *ANXA1* gene on 9q21.13 (Yoon et al., 2024; Granel et al., 2023). The risk allele of this locus was associated with decreased expression of the *ANXA1* gene, and its regulatory role in allergic airway inflammation was confirmed in a mouse model. Joo et al. used Hi-C technology to analyze genetic loci associated with bronchodilator responsiveness and identified a non-coding region within the LINC02220 gene that interacts with the promoter of the *DNAH5* gene (Joo et al., 2022). This finding provides new insights into the genetic basis of bronchodilator response in asthma patients.

A recent study integrated epigenetic markers with genomic interactions in asthma research. Morin et al. identified allergic asthma-related CpGs via whole-genome bisulfite sequencing and *in silico* predictions, designing a custom array targeting functionally relevant loci (Morin et al., 2023). Using two cohorts, they linked CpGs to interacting genes via Promoter Capture Hi-C, and found intermediate methylation CpGs associated with allergic sensitization and eQTM effects. This highlighted 3D chromatin interactions in epigenetic regulation of asthma genes, with such CpGs as potential markers, consistent across populations.

The application of Hi-C technology in COPD and asthma research not only provides new tools for elucidating the regulatory mechanisms of disease-related genes but also offers a new perspective for understanding gene-environment interactions. By analyzing chromosomal interactions, Hi-C technology can identify distal regulatory elements and their interactions with target genes, which is of great significance for understanding the genetic basis of complex diseases. For example, in the studies of COPD and asthma, Hi-C technology has revealed the functions of several non-coding regions associated with disease, which may influence disease pathogenesis by regulating the expression of key genes (Guo et al., 2024). Moreover, Hi-C technology provides new ideas for studying the 3D structure of the genome and its role in disease (Šimková et al., 2024). By analyzing changes in chromosomal conformation, researchers will better understand how the spatial organization of the genome affects gene expression and disease occurrence.

As a powerful genomic tool, Hi-C technology has shown broad application prospects in pulmonary disease research. By revealing the 3D structure of the genome and its regulatory effects on gene expression, Hi-C technology provides a new perspective for understanding the pathogenesis of COPD and asthma. In the future, with further development and application of the technology, Hi-C is expected to play an important role in the genomic research of more pulmonary diseases, offering new strategies for diagnosis, treatment, and prevention.

### 3.2 Lung cancer

Lung cancer is one of the most common malignant tumors globally and the leading cause of cancer-related mortality. It is estimated that there are approximately two million new cases and 1.76 million deaths annually (Thai et al., 2021). Non-small cell lung cancer (NSCLC) and small cell lung cancer (SCLC) are the two primary types of lung cancer, with NSCLC accounting for the majority of cases. In recent years, significant improvements in the prognosis of lung cancer patients have been achieved through advancements in understanding the biological characteristics of lung cancer, the application of predictive biomarkers, and improvements in therapeutic approaches. Important breakthroughs have also been made in the genetic and molecular characterization of lung cancer. Proteomics and genomics studies have revealed the complex molecular features of lung cancer and provided new directions for treatment (Liu et al., 2024; Gao et al., 2019).

The development and progression of lung cancer are closely associated with a variety of genetic alterations, including gene mutations, copy number variations (CNVs), chromosomal rearrangements, and epigenetic modifications. These genetic changes not only affect gene expression but may also promote tumor initiation and progression by altering chromatin structure and gene regulatory networks (Li et al., 2021). In recent years, genome-wide association studies (GWAS) have identified 45 single nucleotide polymorphisms (SNPs) that may be associated with lung cancer susceptibility, most of which are located in non-coding regions (Ji et al., 2020). These SNPs in non-coding regions may regulate gene expression by affecting the availability of transcription factor binding sites, thereby influencing the risk of lung cancer.

Among these advancements, 3D genomics, particularly Hi-C technology, has provided a new perspective for elucidating genomic structural changes in lung cancer. Hi-C technology offers a powerful tool for studying genomic structural changes in lung cancer, especially in revealing the relationship between chromatin structure and gene expression regulation. For example, Ji et al. integrated Hi-C data from lung cancer cell lines with GWAS data from Asian populations to systematically analyze the association between genetic variations in chromatin interaction regions and lung cancer risk. This study identified four new lung cancer susceptibility loci located at 1q21.1, 2p23.3, 2p15, and 17q21.2 ^46^. These loci may regulate the expression of lung cancer-related genes by affecting the availability of transcription factor binding sites. For instance, the *rs9309336* locus may affect the binding site of the transcription factor FOXP1 and regulate the expression of the *CHD1L* gene, thereby influencing the risk of lung cancer.

Li et al. used Hi-C technology to analyze the 3D genomic structure of paired tumor and normal tissue samples from lung cancer patients (Li et al., 2021). The study found significant chromatin structural changes in tumor tissues compared to normal tissues, including compartment switching of A/B compartments, changes in topologically associating domains (TADs), and the formation of enhancer-promoter loops. These changes are closely related to lung cancer-associated signaling pathways, such as *MAPK*, *PI3K-AKT*, *Ras*, and *Wnt* pathways. Additionally, five key genes (*SYT16*, *NCEH1*, *NXPE3*, *MB21D2*, and *DZIP1L*) were identified, which simultaneously appeared in A→B compartment switching, TADs, and chromatin loops in tumor samples, with four of these genes located on the q arm of chromosome 3 (Song et al., 2023). Expression and invasion experiments of these genes suggest that they may play important roles in the occurrence and development of lung cancer. Besides, Adeel et al. used Hi-C technology to detect chromosomal translocations in lung cancer cell lines (e.g., A549) and validated the results using whole-genome sequencing (WGS) and paired-read analysis (Adeel et al., 2022). The study demonstrated that Hi-C data are as reliable as WGS in detecting chromosomal translocations and can be used as an auxiliary tool for studying the 3D structural changes in cancer genomes. These findings support the application value of Hi-C technology in the study of lung cancer genomic structure. Meanwhile, Fan et al. systematically summarized the regulatory mechanisms of PD-L1 from nuclear chromatin remodeling to extracellular presentation and used Hi-C data to reveal a new TAD (chr9: 5,400,000-5,600,000) containing a super-enhancer that drives the synchronous transcription of PD-L1 and PD-L2 in high-expression cancer cells (Fan et al., 2022).

In addition to its applications in basic research, Hi-C technology has also provided new insights into lung cancer treatment. For example, by analyzing the 3D genomic structure of lung cancer cells, potential therapeutic targets and biomarkers can be identified. Wu et al. developed a computational method for quantifying enhancer RNAs (eRNAs) and revealed the association between transcriptional enhancers and lung cancer markers or oncogenes by integrating Hi-C, ChIP-seq, and RNA-seq data. These findings offer new directions for lung cancer treatment based on 3D genomic structure (Wu et al., 2020). Guo et al. used Hi-C technology to develop a computational framework named TARGET for systematically identifying candidate dysregulated genes associated with changes in TAD boundaries and comparing gene expression profiles between SCLC and normal human lung fibroblast cell lines (Guo et al., 2021).

Hi-C technology provides a powerful tool for studying the genomic structure and regulatory mechanisms of lung cancer. By elucidating the 3D genomic structure of lung cancer cells, Hi-C technology not only helps identify new lung cancer susceptibility loci but also reveals the relationship between chromatin structural changes and the occurrence and development of lung cancer. Moreover, Hi-C technology shows great potential in detecting chromosomal translocations and identifying potential therapeutic targets. Future research can further utilize Hi-C technology in combination with multi-omics data to deeply explore the genomic structural characteristics of lung cancer, providing new strategies for diagnosis, treatment, and prognosis.

## 4 Discussion

The advent of high-throughput sequencing technologies has revolutionized our understanding of genomic architecture and function. One such technology, Hi-C enables researchers to analyze the 3D organization of the genome at an unprecedented resolution. Recent studies have highlighted the potential of Hi-C in rapidly identifying therapeutic targets in various contexts, including pulmonary diseases, which encompass a range of pathologies such as COPD, asthma, and lung cancer. Pulmonary diseases are complex and multifactorial, often involving intricate interactions between genetic predispositions and environmental factors. Traditional approaches to identifying therapeutic targets have relied heavily on linear genomic analyses, which may overlook the spatial relationships that are essential for understanding gene regulation and expression in the context of specific diseases (Table 1).

**TABLE 1:** Summarization of Hi-C-based therapeutic targets discovery.

Study	Study model	Hi-C application	Novel targets or site of detection	Epigenetic mechanisms
Mak et al. (2020)	African American children with asthma	Identification of genetic variants associated with lung function (FEV1)	*KITLG* (KIT ligand, also known as *SCF*) on chromosome 12	Hi-C data and expression QTL analysis showed that the variant is associated with increased *KITLG* gene expression, potentially through affecting transcription factor binding sites (e.g., *STAT3* and *IRF1*) regulating *KITLG* expression
Stuart et al. (2020)	Asthma, cystic fibrosis (CF), COPD, and idiopathic pulmonary fibrosis (IPF) loci	Integration of Hi-C, ChIP-seq, and eQTL to predict non-coding region functions	SNPs in regions 19q13, 17q21, and 11p15	Hi-C predicted that these non-coding regions interact with nearby genes and function as enhancers, with CRISPRi validating the regulatory role of some regions on gene expression
Joo et al. (2022)	Minority children with asthma	Identification of genetic variants associated with bronchodilator response (BDR)	*rs35661809* in the *LINC02220* gene region	Hi-C data showed long-range interactions between this region and the *DNAH5* gene promoter in lung tissue, potentially affecting *DNAH5* gene expression
Washington et al. (2022)	African American children and adults with asthma	Identification of African-specific alleles associated with asthma susceptibility	Nine novel variants in the 17q12-q21 region	pcHi-C data narrowed the association to two candidate causal variants, which are associated with T2-low severe asthma traits
Morin et al. (2023)	Nasal epithelial cells from children with allergic asthma	Design of a custom array for high-value CpG sites	CpG sites in the custom Asthma and Allergy array	Hi-C data identified CpG sites associated with gene expression, revealing epigenetic regulatory mechanisms in allergic asthma
Granell et al. (2023)	GWAS of childhood wheezing phenotypes	Identification of genetic loci associated with persistent wheezing	*ANXA1* gene in the 9q21.13 region	Promoter capture Hi-C data identified *rs75260654* as the most likely causal SNP, with the risk allele associated with reduced *ANXA1* expression
Guo et al. (2024)	Human lung epithelial cells	Identification of distal enhancers regulating *HHIP* gene expression	Distal enhancer in the 4q31 region	Hi-C data revealed chromatin interactions between this enhancer and the *HHIP* gene, regulating *HHIP* expression and potentially influencing epithelial-mesenchymal transition (EMT) in bronchial epithelial cells via TGFβ signaling
Ji et al. (2020)	Integration of lung cancer cell lines and GWAS data	Identification of lung cancer susceptibility loci	1q21.1 (rs17160062), 2p23.3 (rs670343), 2p15 (rs9309336), 17q21.2 (rs9252)	Affected transcription factor binding sites (e.g., FOXP1), regulating lung cancer-related gene expression (e.g., *PUS10* and *CHD1L*)
Wu et al. (2020)	Lung cancer samples and cell lines	Analysis of enhancer transcriptional activity	*LcsMYC-1* (lung cancer-specific MYC eRNA-1)	Enrichment of transcription factor binding sites (e.g., *JUNB*, *Hand1-Tcf3*, and *GATA4*), with enhancer RNA (eRNA) associated with oncogene expression
Li et al. (2021)	Lung cancer clinical samples (paired tumor and normal cells)	Analysis of 3D genome structural changes	Copy number variations (CNVs) and point mutations	3D genome structure affects gene expression by altering regulatory chromatin structures
Guo et al. (2021)	Small cell lung cancer (SCLC) cell lines	Identification of dysregulated genes associated with TAD boundary changes	>100 candidate genes (24 validated by NanoString)	Chromatin structural changes at the A/B compartment and TAD boundary levels lead to gene dysregulation
Fan et al. (2022)	Cancer cells with high PD-L1 and PD-L2 expression	Revealing the regulatory mechanisms of PD-L1/PD-L2	New TAD in the chr9: 5,400,000-5,600,000 region	Transcription factors (e.g., *STAT3* and *IRF1*) recruited to the PD-L1 locus, regulating PD-L1 expression
Adeel et al. (2022)	Lung cancer (A549), chronic myeloid leukemia (K562), and acute monocytic leukemia (THP-1) cell lines	Detection of chromosomal translocations	Translocation sites consistent with WGS validation	3D genome structural changes impact gene expression and cancer mechanisms
Song et al. (2023)	Non-smoking lung adenocarcinoma (LUAD) tumors and paired normal tissues	Analysis of chromatin structural changes	216 tumor-specific TADs, 41 enhancer-promoter loops	A/B compartment switching (A→B), with key genes (e.g., *NCEH1*, *MB21D2*, and *SYT16*) involved in tumor development

Hi-C technology allows for the comprehensive mapping of chromosomal interactions, revealing how different regions of the genome communicate with one another. This spatial perspective provides insights into gene regulation that linear sequencing cannot offer. For instance, in the context of pulmonary diseases, Hi-C can uncover interactions between regulatory elements, like enhancers and their target genes, which may be pivotal in the pathogenesis of diseases such as asthma, where inflammatory gene expression is tightly controlled by these interactions.

The application of Hi-C in pulmonary disease research starts with the identification of regions of interest that are associated with disease phenotypes. For instance, researchers can compare Hi-C data from lung tissues of healthy individuals against those from patients with COPD or lung cancer. This comparative approach facilitates the identification of disrupted chromatin interactions that may contribute to aberrant gene expression profiles. Once potential target regions are identified through Hi-C analysis, researchers can prioritize them for further functional validation. This expeditious workflow is particularly advantageous in the context of lung cancer, where traditional methods of target identification can be both time-consuming and costly. By leveraging the spatial data provided by Hi-C, researchers can focus their efforts on specific interactions that are likely to drive disease, thus streamlining the drug discovery process.

Recent studies have demonstrated the utility of Hi-C in identifying therapeutic targets in pulmonary disease. For example, in lung cancer, researchers have mapped chromatin interactions in cancerous *versus* non-cancerous tissues, revealing novel enhancer-promoter interactions that are crucial for the expression of oncogenes (Cigrang et al., 2025). By disrupting these interactions using CRISPR/Cas9 technology, scientists can validate their roles in tumorigenesis and potentially identify new drug targets (He et al., 2024). In the context of asthma, Hi-C has been used to pinpoint chromatin alterations that affect genes known to be involved in airway inflammation (Mak et al., 2020; Granell et al., 2023). These insights enable the identification of novel therapeutic targets that could be explored to develop targeted therapies aimed at alleviating symptoms and reducing airway hyperresponsiveness. To enhance the identification of therapeutic targets, Hi-C data can be integrated with other omics technologies, such as transcriptomics (RNA-seq) and proteomics. For instance, combining Hi-C with RNA-Seq data allows researchers to correlate chromatin interactions with gene expression changes, providing a clearer understanding of how alterations in chromatin organization contribute to disease phenotypes (Lan et al., 2012; Wu et al., 2020).

Moreover, integrating Hi-C with epigenomics, such as DNA methylation and histone modification profiling, can uncover the regulatory mechanisms driving pulmonary diseases. This multi-dimensional approach enables a more comprehensive understanding of the interplay between genomic architecture and gene regulation. While the application of Hi-C in the rapid identification of therapeutic targets in pulmonary diseases is promising, there are challenges to consider. One limitation is the resolution of Hi-C data, which, although significantly improved, may still obscure interactions occurring at small genomic distances (Rao et al., 2014; Jin et al., 2013). Further advancements in Hi-C methodologies, such as single-cell Hi-C, hold the potential to address these limitations by providing insight into chromatin interactions at the single-cell level.

Additionally, the complexity of pulmonary diseases means that a one-size-fits-all approach may not be feasible. Personalized medicine, which tailors treatments based on individual genomic and environmental profiles, will benefit significantly from Hi-C and complementing technologies.

In summary, Hi-C technology represents a powerful tool for rapidly identifying therapeutic targets in pulmonary disease by elucidating the 3D structure of the genome and its impact on gene regulation. By leveraging insights gained from chromatin interactions, researchers can streamline the target identification process, paving the way for novel therapeutic developments. As the field continues to evolve, the integration of Hi-C with other genomic technologies will undoubtedly enhance our understanding of pulmonary diseases and the complex mechanisms underlying their pathogenesis, ultimately contributing to more effective treatments and improved patient outcomes.
